# Inuit-defined determinants of food security in academic research focusing on Inuit Nunangat and Alaska: A scoping review protocol

**DOI:** 10.1177/02601060221151091

**Published:** 2023-01-17

**Authors:** Angus Naylor, Tiff-Annie Kenny, Sherilee Harper, Dorothy Beale, Zahra Premji, Chris Furgal, James Ford, Matthew Little

**Affiliations:** 1 School of Public Health and Social Policy, 8205 University of Victoria, Victoria, British Columbia, Canada; 2 Département de Médecine Sociale et Préventive, 4440 Université Laval, Québec, Quebec, Canada; 3 School of Public Health, 3158 University of Alberta, Edmonton, Alberta, Canada; 4 Libraries, 8205 University of Victoria, Victoria, British Columbia, Canada; 5 Department of Indigenous Studies, 6515 Trent University, Peterborough, Ontario, Canada; 6 Priestley International Centre for Climate, 4468 University of Leeds, West Yorkshire, UK

**Keywords:** food security, food systems, Inuit Nunangat, *Arctic Canada*, *Alaska*, *Canadian Arctic*, *Inuit*, *Inupiat*

## Abstract

**Background:**

Academic research on food security in Inuit Nunangat and Alaska frequently adopts the Food and Agriculture Organization of the United Nations' working definition of food security and Western conceptualisations of what it means to be ‘food secure’. However, in 2014, the Alaskan branch of the Inuit Circumpolar Council (ICC) stated that academic and intergovernmental definitions and understandings ‘are important, but not what we are talking about when we say food security’. The organisation subsequently developed its own conceptualisation and definition: the Alaskan Inuit Food Security Conceptual Framework (AIFSCF), which in 2020 received informal assent by ICC-Canada.

**Aim:**

This protocol establishes a review strategy to examine how well academic research reflects Inuit conceptualisations and understandings of food security, as outlined in the AIFSCF.

**Methods:**

Review structure and reporting will be completed according to adapted RepOrting standards for Systematic Evidence Syntheses (ROSES) guidelines. A comprehensive search strategy will be used to locate peer-reviewed research from Medline, Scopus, Web of Science and the Arctic and Antarctic Regions (EBSCO) databases. Dual reviewer screening will take place at the abstract, title, and full-text stages. Different study methodologies (qualitative, quantitative, and mixed methods) will be included for review, on the proviso that articles identify drivers of food security. An *a priori* coding framework will be applied by a single reviewer to extract data on publication characteristics, methods and article aims. Deductive thematic content analysis will then identify the frequency and precedence afforded within literature to the drivers and dimensions of food security identified by the AIFSCF.

## Background

### The concept of ‘food security’

Food security remains a multifaceted, contested and pluralistic concept (Jones et al., 2013; Renzaho and Mellor, 2010). As of 1999, over 200 definitions of what it meant to be ‘food secure’ were published in academic and grey literature (Smith et al., 1992; Hoddinott, 1999), and following calls for the incorporation of principles such as agency and sustainability, this number has only increased over the last three decades (Renzaho and Mellor, 2010; Clapp et al., 2022; Berry et al. 2015). Early designations of food security, established following the 1974 United Nations World Food Conference, placed a strong focus on the international context of food, the production and supply of food, and global trade networks; specifically, the ‘*availability* at all times of adequate world supplies’ (Maxwell, 1996; Candel, 2014). With time, the hegemonic concept of food security has further developed. Most notably with the creation (and subsequent iterations) of a definition of food security at the 1996 World Food Summit, which includes a further three core ‘dimensions’: ‘*economic and physical access’, ‘utilisation’,* and ‘*stability over time’*. The FAO 1996 definition and its amendments have become the most widely accepted and used in policy and academic literature. This framing of food security has incorporated a greater focus on entitlements and explores how ‘livelihoods security’, the right to food, preference, and sustainability can affect an individual or peoples’ ability to lead a healthy life with access to culturally relevant foods (Sen, 1981; Jones et al., 2013; Candel, 2014; FAO, 2021). Below, we include the most recent iteration, published as part of the Food and Agriculture Organization of the United Nations' (FAO) annual 2021 *State of Food and Nutrition in the World* report:

Food security is ‘[a] situation that exists when all people, at all times, have physical, social and economic access to sufficient, safe and nutritious food that meets their dietary needs and food preferences for an active and healthy life. Based on this definition, four food security dimensions can be identified: food availability, economic and physical access to food, food utilization and stability over time.’ (FAO, 2021).

A range of measures and indicators are available to assess food security prevalence, such as the United States Department of Agriculture's (USDA) Household Food Security Survey Module and its six-item short form iteration (see USDA, 2012; Statistics Canada, 2022). Quantifying food insecurity in this way has considerable utility: data from food security surveys have been used as a means of positive empowerment by a number of Indigenous and minority rights groups in North America, providing an opportunity to expropriate a tool, legitimated and developed by national governments, to evidence structural violences (e.g., colonialism) or the ineffectiveness of current food policies (e.g., NFSC, 2014; St Germain et al. 2019; ITK, 2021). However, the nuance and complex make-up of many Indigenous food systems in the United States and Canada, combined with the numerous, pluralistic understandings of what it means to be ‘food secure’, can also make tracking and developing an understanding of food security challenging (Power, 2008; Jones et al., 2013; Council of Canadian Academies, 2014; ITK, 2021).

This issue is particularly pronounced for Inuit, whose food systems – while entangled with economic systems and global capital markets as a result of historic and modern-day colonialism (e.g., neoliberal capitalism, globalisation) – continue to prioritise and value the land-based harvesting and foraging of country foods (Gombay, 2005; Wenzel, 2019; Naylor et al., 2021a). These ‘mixed’ or ‘dual’ food systems are oriented towards far more than nutrition, economic access to food, or the role of food in ensuring a healthy life (Papatsie et al. 2013; Ready, 2016; Gombay, 2005; ICC-Alaska, 2015a, 2020; Stephenson, 2021). Inuit food systems are a means of preserving culture and spirituality. The practice of sharing and hunting country foods is considered a way of ensuring continuity with a pre-colonial past and research has also linked land-based activities and harvesting with improved mental health and well-being (Condon et al. 1995; ICC-Alaska, 2020; Cunsolo Willox et al. 2013; Sawatzky et al., 2019).

### Food security in Arctic North America, Inuit Circumpolar Council (Alaska)'s definition and conceptualisation

The issue of food security has come to dominate academic research and public policy spheres in Inuit Nunangat (the Inuit homeland in Canada) and Alaska over the last two decades. In part due to its intersection with broader issues relating to colonialism, Indigenous Peoples’ health, and climate change (e.g., Chan et al. 2006; Ford and Berrang-Ford, 2009; Council of Canadian Academies, 2014; ITK, 2021). However, in 2014, the Inuit Circumpolar Council (ICC) (Alaska) (2014, p. 4) noted that contemporary definitions of food security and the application of food security approaches used by Western researchers and international organisations ‘do not necessarily reflect the Alaskan Arctic environment or its food webs’. Subsequently, they have stated that ‘nutritional value, calories and money needed to purchase food…[are] important, but not what we are talking about when we say food security’ (ICC-Alaska, 2015b, p. 8). Wider critiques of hegemonic perspectives on food security have also pointed to the myopicism of many conceptualisations, which recognise the existence of inequalities but fail to attribute colonial, imperial, or capitalist power relations as socioeconomic and political root causes, and a lack of nuance regarding social relationships, culture, and intra-community dynamics in non-market driven food environments (e.g., gender, age) (Stephenson, 2021; Devereux, 2001). In asserting the need for clarification on what food security means to Inuit, and arguing for a conceptual departure from the more well-established FAO 1996 definition and its subsequent iterations, ICC-Alaska outlined their intention to create the ‘Alaskan Inuit Food Security Conceptual Framework’ (AIFSCF) (ICC-Alaska, 2014).

Published in 2015, the AIFSCF (ICC-Alaska, 2015a) details a new definition and conceptualisation of food security, co-developed by 146 representatives from 15 Alaskan Inuit communities (‘Inupiat of the North Slope, Northwest and Bering Strait; the St Lawrence Yupik; and the Central Yup’ik and Cup’ik of the Yukon-Kuskokwim region’) and a 12-member Food Security Advisory Committee. The AIFSCF incorporates six dimensions of food security (*Availability*, *Inuit Culture*, *Decision-Making Power and Management*, *Health and Wellness*, *Stability* and *Accessibility*) and identifies 58 specific drivers (e.g., *Mental Wellness*, *Burden of Conservation*, *Institutional Racism*) that can contribute to and/or mitigate against food insecurity in Inuit Nunangat and Alaska (see Supplementary Materials 1). (For the purposes of the ICC report, food insecurity is considered the inverse of food security; ICC-Alaska, 2015a). The AIFSCF also emphasises the need for a greater combination of the concepts of food security and food sovereignty (i.e., the right to food, and the right to self-determination in food systems) (see Declaration of Nyéléni, 2007; ICC-Alaska, 2020). It contends that ‘without food sovereignty, food security will not exist’ (ICC-Alaska, 2015a, p. 5) and establishes the groundwork for a more politicised food environment. The AIFSCF is therefore distinct from the FAO (2021) definition, which instead has four pillars (*Access, Availability, Stability* and *Utilisation*), does not make an explicit link to food sovereignty and does not identify specific factors that affect food security.

Inuit Food Security is the natural right of all Inuit to be part of the ecosystem, to access food and to care-take, protect and respect all of life, land, water, and air. It allows for all Inuit to obtain, process, store, and consume sufficient amounts of healthy, nutritious, and preferred food – foods Inuit physically and spiritually crave and need from the land, air, and water. These foods provide for families and future generations through the practice of Inuit customs and spirituality, languages, knowledge, policies, management practices, and self-governance. It includes the responsibility and ability to pass on knowledge to younger generations, the taste of traditional foods rooted in place and season, knowledge of how to safely obtain and prepare traditional foods for medicinal use, clothing, housing, nutrients and, overall, how to be within one's environment. It means understanding that food is a lifeline and a connection between the past and today's self and cultural identity. Inuit food security is characterized by environmental health and is made up of six interconnecting dimensions: 1) Availability; 2) Inuit Culture; 3) Decision-Making Power and Management; 4) Health and Wellness; 5) Stability; and 6) Accessibility. This definition holds the understanding that without food sovereignty, food security will not exist. (ICC-Alaska, 2015a).

Accompanying the AIFSCF, ICC-Alaska also published a technical report, which outlines the specific information and criteria that are required to inform each driver and dimension of food security they identify (ICC-Alaska, 2015b) (Supplementary Materials 1). The technical report provides a key context to both the meaning behind each dimension and driver, and the reasons why each was chosen and is of importance to Inuit understandings of, and requirements for, food security (ICC-Alaska, 2015b). In 2020, the ICC-Alaska (ICC-Alaska, 2014) definition was recognised and applied by stakeholders from ICC-Canada as part of the ‘Food Sovereignty and Self Governance’ report (ICC-Alaska, 2020).

This protocol outlines the process of creating a database of food security literature in Inuit Nunangat and Alaska and describes methods that will be used as part of a review that will examine how academic research has reflected Inuit conceptualisations and understandings of food security over the past two and a half decades, as outlined in the AIFSCF. The authors have chosen to include Inuit represented by ICC-Alaska and ICC-Canada living in Arctic North America (i.e., Inuit, Inupiat, Yupik, Yup’ik and Cup’ik), as these are regions that have approved, formally or informally, the definition provided in the AIFSCF. Applying the six dimensions and 58 determinants of food security identified by ICC-Alaska (2015b) as its *a priori* framework, the review developed from this protocol will assess how past research addressing determinants of food security in Inuit Nunangat and Alaska has captured the drivers defined by ICC. By extension, it will examine how representative this research has been of Inuit-defined understandings of what it means to be food secure.

## Methodology

Owing to the multidisciplinary nature and range of scholarship addressing the topic of food security in Inuit Nunangat and Alaska, which spans research situated in sociology, anthropology, environmental contaminants, and the ‘human dimensions of climate change’ (e.g., Ford, 2009; Brubaker et al. 2011; Naylor et al. 2021b; Fazzino and Loring, 2009; Beaumier et al. 2015) through to epidemiological and dietetics studies (e.g., Sharma et al. 2013; Huet et al. 2012; Egeland et al. 2010, 2011; Kenny et al. 2018), it was decided that a modified scoping review utilising deductive thematic analysis was best suited to answer the research question. Scoping reviews are an effective means of systematically identifying key characteristics relating to specific concepts in bodies of literature with considerable methodological diversity; establishing how a specific term or concept is used, by whom and for what purpose; for identifying and mapping gaps in knowledge; and for developing recommendations for future research approaches (Anderson et al., 2008; Munn et al., 2018; Micah et al., 2015; Berrang-Ford et al.*,* 2015).

### Stakeholder engagement

The scope and objectives of the review have been refined through discussions with academic experts in the field of food security and nutrition, and a University of Victoria research librarian. As part of the process, both the Canadian and Alaskan chapters of the Inuit Circumpolar Council (ICC-Alaska, ICC-Canada) and Inuit Tapiriit Kanatami (ITK) will be engaged to ensure that Inuit priorities for research are reflected, and to maintain the integrity and accuracy of the results prior to publication.

### Research question, objectives and hypothesis

The primary research question for this scoping review is as follows:

- To what extent does academic research on food security consider Inuit conceptualisations of what it means to be food secure, based on the six dimensions outlined in the AIFSCF?

Secondary research questions that will be explored are:

- How well has past research addressed determinants of food security captured by the ICC-defined AIFSCF drivers?
- What has past research missed/failed to examine in the ICC AIFSCF definition thus far?

The hypothesis for this scoping review is that academic research on food security in Inuit Nunangat and Alaska will identify the drivers outlined in the AIFSCF that fall within dimensions of food security that overlap with the FAO (2021) definition (e.g., Accessibility, Stability, Availability). Conversely, it is anticipated that there will be a lack of identified drivers that correspond to the AIFSCF-specific dimensions of food security (e.g., Inuit Culture, Health and Wellness, Decision-Making Power and Management).

### Methods

#### Search methodology and screening process

Comprehensive searches will be developed with a professional librarian to identify peer-reviewed literature in four bibliographic databases: Medline (via Ovid), Scopus, Web of Science and the Arctic and Antarctic Regions Database (EBSCO). To locate articles published between 1 January 1996 and 31 December 2022, we will create a comprehensive search strategy incorporating subject headings (where available) and keywords, and using Boolean and other available search operators. No language restrictions will be placed on the search. 1 January 1996 was chosen as the starting date for the literature search as this was the year in which the seminal FAO (1996) definition of food security and its ‘four pillars’ was developed. This also covers the point (2004) before and after which both Canada and the United States monitored food security at the household scale through the use of standardised scales (the USDA Household Food Security Survey Module) (Tarasuk et al. 2018). The authors understand and recognise that ICC represents all circumpolar Inuit and acknowledge that there may be a diversity of perspectives on this issue of food security among Kalaallit, Chukchi, and other peoples represented by the ICC based upon the experience living under different settler governments and administrations.

The search string includes three groups of search terms, focusing on geographical area (where the ICC-Alaska definition has been endorsed), thematic focus (the issue of food security), and population (Inuit) (Box 1). The string was adapted from previous systematic reviews focusing on Indigenous Peoples’ food security and nutrition (e.g., Luongo et al., 2020; Kenny et al., 2020; Little et al., 2021), with modifications made based on the authors’ *a priori* knowledge on the subject, the specific thematic focus the of review, through using database thesauruses (e.g., MeSH (PubMed)), and through consultation with the departmental librarian at the School of Public Health and Social Policy at the University of Victoria. Further refining of the search string also took place following interactive scanning of the full text of five seed papers by the librarian, which were selected from results in the first iteration of the search string in Medline. ‘Seed papers’ describe influential articles, typified by a high citation count and ‘centrality’ (number of links with other publications and authors), within a field of study (Wang et al. 2021). As per Booth (2008), interactive scanning is suitable for users who are not experts in a specific topic area. It requires starting from a broad concept (i.e., Inuit food systems) and then refining search terms as this concept becomes progressively clearer to the reader based on the reading of seed papers. In doing so, redundant search terms are removed (through discussion with other paper authors) and relevant terms either retained or added.

Box 1. Medline (via Ovid) draft search strategy.

**Table:** 

#	Query	Results from 30 June 2022
1	food supply/ or famine/ or food deserts/ or food insecurity/ or access to healthy foods/ or food security/	15,859
2	((food* or diet*) adj5 (secur* or insecur* or scarc* or sovereign* or availab* or access* or stabil* or stable* or prefer* or qualit* or traditional* or country* or network* or system* or poverty or poor or desert* or market or "store-bought" or utili?ation*)).tw,kf,ot.	107,692
3	(diet* or nutrition* or malnutrition* or malnourish* or nutrient* or hunger*).tw,kf,ot.	1,037,167
4	1 or 2 or 3	1,086,337
5	Arctic Regions/	7256
6	(((Canada or Nunavut or Nunatsiavut or Nunavik or Inuvialuit Settlement Region or Alaska* or Far-North or North or Northwest Territories or Newfoundland or Labrador) or Inuit Nunangat or ((North America* or canad* or united states) adj2 Arctic)).tw,kf,ot.	290,401
7	exp Nunavut/ or Nunavut.mp. or Eastern Arctic.mp. or Alert Bay.mp. or Alexandra Fiord.mp. or Amadjuak.mp. or Aquiatulavik Point.mp. or Arctic Bay.mp. or Arviat.mp. or Baffin Island.mp. or Baker Lake.mp. or Bathurst Inlet.mp. or Belcher Islands.mp. or Bylot Island.mp. or Cambridge Bay.mp. or Iqaluktuttiaq.mp. or Cape Dorset.mp. or Cape Dyer.mp. or Cape Smith.mp. or Charlton Depot.mp. or Chesterfield Inlet.mp. or Clyde River.mp. or Coral Harbour.mp. or Craig Harbour.mp. or Dundas Harbor.mp. or Ellesmere Island.mp. or Ennadai.mp. or Eskimo Point.mp. or Fort Conger.mp. or Fort Hope.mp. or Fort Ross.mp. or Gjoa Haven.mp. or Grise Fiord.mp. or Hall Beach.mp. or Hazen Camp.mp. or Igloolik.mp. or Ikaluit.mp. or Iqaluit.mp. or Isachsen.mp. or Kekerten.mp. or Kimmirut.mp. or King William Island.mp. or Kipisa.mp. or Kitikmeot o r Kivalliq.mp. or Kivitoo.mp. or Kugaaruk.mp. or Kugluktuk.mp. or Maguse River.mp. or Nanasivik.mp. or Nottingham Island.mp. or Nuwata.mp. or Padlei.mp. or Padloping Island.mp. or Pangnirtung.mp. or Perry Island.mp. or Pond Inlet.mp. or Port Burwell.mp. or Qoloqtaaluk.mp. or Qikiqtarjuaq.mp. or Rankin Inlet.mp. or Read Island.mp. or Repuilse Bay.mp. or Resolute Bay.mp. or Resolution Island.mp. or Sanikiluak.mp. or Taloyoak.mp. or Tanquary Camp.mp. or Tavani.mp. or Thom Bay.mp. or Umingmaktok.mp. or Victoria Island.mp. or Wager Bay.mp. or Whale Cove.mp. or Eastern Arctic.mp. or ((Lupin or Polaris or Eureka or Fullerton) and Canad*).mp.	1117
8	5 or 6 or 7	1295,331
9	4 and 8	18,310
10	(Inui* or Eskimo* or Inuq or Inuk* or Inuvial* or Inupia* or Nunav* or Alaska Native* or Yupik or Cupik).tw,kf,ot.	7569
11	inuits/ or "american indians or alaska natives"/ or alaskan natives/	5145
12	10 or 11	9219
13	9 and 12	820

*Note:* In order to develop a search string that would capture all articles potentially relevant to Inuit, it was necessary to recognise the unethical terminologies that have been (and occasionally continue to be) used to describe Indigenous Peoples in academic research (Tuhiwai Smith, 1999; Younging, 2018). One of the terms in this list is no longer considered appropriate for use with Inuit communities and is frequently considered offensive. The term was included here to capture literature from previous decades prior to discontinuation of the term and does in no way reflect the authors’ beliefs about or relationships with Indigenous Peoples. If an article could be considered racist, harmful or unethical, it will be excluded from review. We also note that Medline uses the ‘MeSH’ search term ‘Inuits’, which is an incorrect pluralisation of ‘Inuit’ (already plural); although grammatically incorrect, it has been retained to find the greatest number of articles in the search.

To be included in the review, articles derived from the search string will be independently screened and assessed for eligibility at two stages based on a number of inclusion and exclusion criteria (Box 2). Screening in the first stage will use two independent reviewers. Disagreement at the first stage of screening will result in automatic inclusion into the second stage. Disagreement at the second stage will be resolved by consensus or, failing that, mediation by a third reviewer with a majority vote. Inclusion/exclusion criteria will include publication between 1 January 1996 and 31 December 2022, being original research articles accessible from either the University of Victoria (Canada) or the University of Leeds’ (United Kingdom) library subscriptions, and having an explicit focus on food security in Inuit Nunangat or Alaska while also identifying determinants of food security. The first stage of screening will occur following the import of articles into DistillerSR – which will be used for organising the papers extracted from databases – where duplicates will be removed, and titles, keywords and abstracts will be scanned and assessed for eligibility, in addition to their publication details (e.g., date). Articles remaining after this stage will then be subject to a second round of screening, with papers read in their entirety and inclusion/exclusion criteria re-applied. The Kappa score will be extracted from DistillerSR and reported for both the first and second stages of screening to indicate the level of agreement between reviewers. The list of remaining articles will then be supplemented with relevant papers derived from snowballing (scanning titles of literature in reference lists of included articles), in addition to the titles in bibliographies of reviews identified during the review process (Hirt et al., 2020).

Box 2. Criteria for inclusion and exclusion from the scoping review.

**Table:** 

Inclusion criteria	Exclusion criteria
Published after 1 January 1996.	Published after 31 December 2022.
Journal article comprising original research.	Abstracts, systematic or scoping reviews, conference proceedings, books, book chapters, white papers and non-empirical research.
Article is an example of empirical research and outlines the methodology used within the study.	Article is conceptual or methodological in nature, without significant empirical research.
Article focuses on Inuit living in either Inuit Nunangat or Alaska.	Article does not focus on Inuit living in either Inuit Nunangat or Alaska.
Study has an explicit focus on food security (and aims to identify determinants/factors affecting food security).^†^	Study does not focus on food security (and does not aim to identify determinants/factors affecting food security).^†^

^†^ The bracketed component of the inclusion/exclusion criteria will only be applied in the second round of screening.

#### Coding framework and data synthesis

A preliminary evaluation rubric has been developed in the form of an electronic codebook, adapted from the application of previous examples to subject areas focusing on Arctic Indigenous populations (e.g., MacDonald et al., 2013), and from the 58 food security drivers established in the ICC-Alaska report (Box 3). As per Granheim (2020), the rubric will be piloted by two reviewers on a random sample (10%) of studies that are retained for review, after which point it will be amended to improve its efficacy and materiality. Any disagreement in amendments to the rubric will be mediated via a third reviewer, with the aim of developing a consensus. Failing that, the third reviewer will cast a vote to make a majority decision. Following amendment, the codebook will then be applied in its final form to all full-text articles by a single reviewer, except for questions 18 and 19 which will be scored by two reviewers (Box 3). The second reviewer will also independently review a sample (10%) of the total articles in their entirety to ensure accuracy.

Box 3. Draft evaluation rubric/codebook for the scoping review.

**Table:** 

**Authorship/Publication Details:**			**Study Area/Sample**		
First author	1		Country and region of study	7	*(Canada, or Alaska? Which territory or region?)*
Year	2		Sample size	8	
Country of first authorship	3		Study population:	9	*Are gender, age or other factors considered?*
Title:	4				
Journal	5				
Engagement of Indigenous organisations or communities of study	6	*Are any authors affiliated with Indigenous organisations or are Indigenous organisations authors themselves? Are communities of study authors? To what extent are they involved in the paper, are their activities (data analysis, writing, etc.) listed in an author contributions section?*			
**Methodology and Methods:**					
Aims/Objectives:	10	*What is the stated objective/objectives of the publication?*			
Study timescale	11	*How long did the study run for?*			
Research Methods:	12	*e.g., Semi-structured interviews, HFSSM, food diaries etc.*			
Food security measured?	13	*Y/N*			
Inuit Knowledge:	14	*Does the study incorporate approaches/methods that integrate Inuit Knowledge or Inuit voices and perspectives?*			
CBPR:	15	*Could the study be considered to have incorporated approaches/methods that could be participatory or community-based in scope?*			
Indigenous methodology/Inuit Qaujimajatuqangit:	16	*Does the paper state that it is using an Indigenous methodology or is incorporating aspects of Inuit Qaujimajatuqangit in its methodology/methods?*			
**Food security**					
Does the article directly state a definition or definitions of food security, if so which?	17	*Is the FAO 1996 definition of food security or subsequent iterations or a different one? Does the paper develop its own definition?*			
ICC dimensions of FS identified:	18	*e.g., Inuit Culture, Accessibility, Health and Wellness*			
ICC determinants of FS identified (+ level of emphasis)	19	*e.g., Hope, Physical Safety*			
Future research directions/limitations:	20	*Does the article make recommendations for future research or note potential limitations, if so what are they?*			
Consequences of food insecurity:	21	*Does the article attribute impacts, if so what are they?*			
**Other:**					

*Note:*

Using the codebook, we will extract descriptive statistics, such as the publication trends of the articles (e.g., year, geographic location, authorship), study design (e.g., quantitative, qualitative or mixed-methods; longitudinal or cross-sectional) and methods used (e.g., semi-structured interviews, Household Food Security Survey module and focus groups). The context of each article's findings will then be examined (e.g., descriptive notes as to their aims and key findings), in addition to any applied themes or concepts relating to food security (e.g., inclusion of FAO (2021) definition of food security or previous iterations). To add legitimacy to possible critiques between Western/hegemonic and Indigenous perspectives in academic research, and to account for the increasing diversity of perspectives brought by Indigenous scholars to this research area, the papers will also be assessed for Indigenous engagement. Specifically, this will be done through a) identifying whether any Indigenous organisations or communities (e.g., the ‘Community of Arviat’) were involved in the publishing process and whether they were involved in the conceptualisation, paper writing or data analysis of research (listed in ‘author contributions’ sections), and b) through identifying whether a paper stated that it was using an Indigenous methodology or the principles of Inuit worldviews (e.g., *Inuit Qaujimajatuqangit)* (Box 3). Finally, deductive thematic content analysis (Neuendorf, 2018; Kiger and Varpio, 2020) will be used to code whether each article identified or explored any of the 58 drivers of food security delineated by ICC-Alaska (2015a, 2015b), which will then be grouped by their respective dimension (e.g., ‘*Economics (Cash Economy*)’ (driver) -> ‘*Accessibility’* (dimension)). The analysis will be semantic, rather than latent/interpretive: the reviewer will look for explicit mention of the ‘dimensions’ and ‘drivers’ identified by the AIFSCF within peer-reviewed literature, as opposed to identifying areas where papers allude to or imply the importance of certain determinants (Braun and Clarke, 2006). This is based on the fact that much food security research has the stated impact of ‘informing policy’ (e.g., Lardeau et al. 2011; Chan et al. 2006; Egeland et al. 2011), which, according to Bulmer (2021), requires the explicit rather than implicit communication of concepts and findings to stakeholders, whereby they cannot be expected to ‘read between the lines’. In addition to the number of articles that identify a specific driver and dimension, the codebook will also establish the extent to which each driver is discussed within the articles. This component of the rubric (questions 18 and 19, Box 3) will require two reviewers, both of whom will act as scorers. Upon the identification of a driver within a dimension, each reviewer will assign a value weighting to said driver. The average scores of both reviewers will be used to generate a final value. A value of ‘one’ attached to the code would mean that the driver was mentioned or identified in passing, while ‘four’ might indicate that the identified driver was the explicit focus of the research article or sections within it (see Box 3 for a full draft evaluation rubric, and Supplementary Materials 2 for weighting table).

Review structure and reporting will be completed according to an adapted version of the RepOrting standards for Systematic Evidence Syntheses (ROSES) guidelines (Haddaway et al. 2018). ROSES is an alternative to the PRISMA or PRISMA-scoping guidelines, developed specifically for the reporting of syntheses of non-medical, qualitative, narrative, quantitative or mixed methods articles. ROSES is especially relevant for a scoping review of this type, as it places a greater emphasis on limitations to the viability of reviews beyond simply exploring the risk of bias (the latter is not ordinarily required for scoping reviews), and is well suited to a review with a deductive framework (Haddaway et al. 2018).

#### Ethical statement and public involvement

This research will be a scoping review and will therefore not involve the input of the public, nor any research participants. As such, it does not require ethical approval. Due to the nature of the topic, permission and assent for this review, in addition to consultation throughout the process, will be sought from the ICC and ITK. Prior to the review's full publication, the results will be shared with both organisations for their input and comment.

## Limitations

A number of possible limitations arise in this study that should be acknowledged. Although the methodology was developed by experts working across a range of disciplines in food security and nutrition (e.g., epidemiology, dietetics, sociology) and a university librarian, the *a priori* knowledge of the authors may bias the study towards certain literature. In addition, due to the multifaceted nature of social–ecological systems research, especially when it comes to country foods, there is a range of research in the areas of wildlife biology and ecology, agrostudies, food and resource economics, and food and public policy that could be considered relevant but does not explicitly state the implications of its findings for Inuit food security and will therefore not be included for review. Although the meaning underlying food security drivers identified by ICC-Alaska is well-defined and outlined in the technical report that accompanies the AIFSCF, it is accepted that this may not represent all perspectives or worldviews vis-à-vis the diverse issue of food security in Inuit Nunangat and Alaska. Moreover, as the reviewers themselves do not identify as Inuit, further interpretation beyond the statements of what constitutes specific determinants and dimensions within the technical report is not deemed appropriate. Finally, though this paper addresses the topic of how Inuit-identified determinants of food security have been represented in academic research, and much of this is presented in the format of peer-reviewed literature, this review will not cover grey literature, where Inuit-led research may be more common.

## Supplemental Material

sj-docx-1-nah-10.1177_02601060221151091 - Supplemental material for Inuit-defined determinants of food security in academic research focusing on Inuit Nunangat and Alaska: A scoping review protocol Click here for additional data file. Supplemental material, sj-docx-1-nah-10.1177_02601060221151091 for Inuit-defined determinants of food security in academic research focusing on Inuit Nunangat and Alaska: A scoping review protocol by Angus Naylor, Tiff-Annie Kenny, Sherilee Harper, Dorothy Beale, Zahra Premji, Chris Furgal, James Ford and Matthew Little in Nutrition and Health
