# Supplementary material for: Inuit-defined determinants of food security in academic research focusing on Inuit Nunangat and Alaska: A scoping review protocol
Source: Nutr Health. 2023 Jan 17;29(2):175–83. doi: 10.1177/02601060221151091 (PMC10114254; doi:10.1177/02601060221151091)
Supplement: sj-docx-1-nah-10.1177_02601060221151091 - Supplemental material for Inuit-defined determinants of food security in academic research focusing on Inuit Nunangat and Alaska: A scoping review protocol [file sj-docx-1-nah-10.1177_02601060221151091.docx]

**Supplementary Materials 1:**

Examples of dimensions and drivers of food security, with indicators, as identified by ICC-Alaska (2015b).

| Dimension | Driver | Given Indicator/Question/Context |
| --- | --- | --- |
| Inuit Culture | Physical Safety | - Are navigation skills passed between generations - Are navigation skills adequate to support safety - Are processing and storing processes to ensure physical safety passed between generations - What are food safety procedures |
| Inuit Culture | Passage of Knowledge | - Is the passage of knowledge between Elders and younger generations supported throughout a given area to the satisfaction of Inuit within a given area - Is the passage of knowledge between Elders and younger generations supported within the education system to the satisfaction of Inuit within a given area |
| Inuit Culture | Sharing Systems | - Identify past and current sharing systems - Is the sharing system supported within regulations to the satisfaction of Inuit within a given area - How is food distributed within the village, regionally, in-state and nationally |
| Availability | Being Able to Eat What Has Been Gathered From Last Season | - Do activities support the gathering and storing of food to the satisfaction of Inuit within a given area? |
| Availability | Variety (Biodiversity) | - Is diversity and variety maintained within a given area? - Do activities inside and outside a given area support the maintenance of diversity and variety within a given area? - How does change in variety affect hunting strategies? |
| Decision-making Power and Management | Burden of Pollution | - Is there a strong polluter-pays policy? - Are those responsible for the generation of pollution expected to change their behaviour? |
| Decision-making Power and Management | Taxation without Representation | - Do political leaders hold adequate understanding of Inuit culture and overall Arctic ecology? |
| Decision-making Power and Management | Institutional Racism | - Do current interpretations of policies and implementation of regulations reflect any level of institutional racism according to Inuit of a given area? |
| Health and Wellness | Air, Land and Water Pollution | - Identify key contaminant pathways within a given area - What are possible impacts of contaminants within a given area, e.g., what are the life cycles within a given area - Is communication of contaminants enacted to the satisfaction of Inuit within a given area - Is contaminant information shared with a given area in a way that they will be able to utilize the information to the satisfaction of Inuit |
| Health and Wellness | Nutrition | - Is the variety of food within a given area adequate to support nutritional needs? - Are there food and nutrition services? - Knowledge about healthy eating and traditional foods |
| Health and Wellness | Housing Structures | - Were Elders and their knowledge used to inform placement and technical environmental processes in designing and constructing structures of a given area? - Do housing structures support cultural activities related to food security, such as drying food to the satisfaction of Inuit within a given area? - Do housing structures provide adequate ventilation systems and are they energy efficient? |
| Accessibility | Ability to Access Healthy Animals, Plants, Fish, Ice, Water, etc. | - Is the knowledge needed available to the satisfaction of Inuit within a given area? - Are outside activities impacting accessibility? - Are non-Inuit cultural activities and development impacting accessibility? - Are there regulatory, statutory or constitutional barriers to access and utilization of traditional Inuit resources. |
| Accessibility | Access to Traditional Territories | - Is there adequate access to traditional food-gathering areas? - Is there adequate access to traditional areas used to transfer knowledge? - Is there adequate access to traditional sacred areas? |
| Accessibility | Economics (Inuit Economy) | - Is trading, sharing and giving supported within a given area to the satisfaction of Inuit within a given area? - Community and household demographics; how many people in the community, how many homes, how many households provide for others (super households)? - Community resources and assets (e.g. community freezers); how many programs support the collection of traditional foods? - How is food distributed within the village, regionally and nationally? |
| Accessibility | Economics (Cash Economy) | - Do current subsidies support self-efficiency - Are the government subsidies provided culturally appropriate? |
| Stability | Ecological Stability | - Identify change resulting from industrialisation - Identify change in flora and fauna - Are current management practices used to ensure future generation and overall ecosystem health? - Do management practices and policies support the mitigation causes of large changes, such as climate change? |
| Stability | Inuit Mental Security | - How many weather-related disasters occured within a given period? - Those already vulnerability will be more vulnerable as more changes occur. |

**Supplemental Materials 2:**

Method for identifying precedence/weighting given to driver of food security in reviewed paper

| Rating | Precedence | Criteria |
| --- | --- | --- |
| 1 | Limited | - Driver is identified, but it is mentioned with little exploration or expansion. |
| 2 | Minor | - Driver identified with some expansion. |
| 3 | Moderate | - Driver identified with considerable expansion and analysis. |
| 4 | Major | - Driver is identified within the paper and is discussed at great length, being either the main focus of the paper, or being the focus of a specific section (i.e. the discussion, or a headed sub section). |
